# Osteogenic Potential of Human Umbilical Cord Mesenchymal Stem Cells on Coralline Hydroxyapatite/Calcium Carbonate Microparticles

**DOI:** 10.1155/2018/4258613

**Published:** 2018-09-05

**Authors:** A. G. E. Day, W. R. Francis, K. Fu, I. L. Pieper, O. Guy, Z. Xia

**Affiliations:** ^1^Institute of Life Science, Swansea University Medical School, Swansea SA2 8PP, UK; ^2^Department of Orthopaedic Surgery, The First Affiliated Hospital of Hainan Medical College, Hainan, China; ^3^College of Engineering, Swansea University, Swansea SA2 8PP, UK

## Abstract

Coralline hydroxyapatite/calcium carbonate (CHACC) is a biodegradable and osteoconductive bone graft material with promising clinical performance. CHACC has been shown to support proliferation and osteogenic differentiation of human bone marrow mesenchymal stem cells (MSCs) *in vitro* and demonstrated to work as a functional scaffold for bone formation *in vivo.* Umbilical cord matrix is a more accessible and abundant tissue source of MSCs, but its osteogenic capacity in comparison to human bone marrow when cultured on CHACC has not yet been demonstrated. In this study, we assessed the osteogenic differentiation capacity of human MSCs, isolated from bone marrow and umbilical cord matrix and characterised by flow cytometry, when cultured on 200–300 *μ*m CHACC granules. The 3D cultures were characterised by brightfield and scanning electron microscopy (SEM). Osteogenic potential was assessed by immunocytochemistry and qPCR for key markers of bone differentiation (alkaline phosphatase, runx2, type I collagen, and osteocalcin). By day 1, the MSCs had enveloped the surface of the CHACC granules to form organoids, and by day 7, cells had proliferated to bridge nearby organoids. Extracellular matrix deposition and osteogenic differentiation were demonstrated by MSCs from both tissue sources at day 21. However, MSCs from bone marrow demonstrated superior osteogenic differentiation capability compared to those from umbilical cord matrix. In conclusion, it is possible to culture and induce osteogenic differentiation of umbilical cord matrix MSCs on CHACC. Further research is required to optimise the osteogenicity of umbilical cord matrix MSCs to release their full potential as a readily available, accessible, and abundant tissue source for bone tissue engineering.

## 1. Introduction

Of the diverse range of scaffolds available for use in maxillofacial surgery and dentistry, autografts have been reported to be the “gold standard” with respect to bone grafting procedures [1]. However, harvesting of autografts, usually from the iliac crest, requires surgical intervention, which is associated with additional risks of blood loss, infection, and morbidity, and supply is limited [2, 3]. Other types of grafts include allografts and xenografts, but these can cause an immunological reaction and be rejected by the recipient [3]; so, it is vital to identify suitable alternative materials.

Synthetic biomaterials, such as hydroxyapatite, tricalcium phosphate ceramics and cements, and bioglass, are alternative sources for bone graft substitutes. However, these synthetic biomaterials do not mimic the architecture, porosity, and organic components of the natural bone and are not optimal in regard to biodegradation and host tissue integration or practical to implant or inject. Naturally occurring coral exoskeleton has a porous architecture that is similar to the human trabecular bone [4]. Since its main composition is calcium carbonate, a hydrothermal technique was developed to completely convert the calcium carbonate to be coralline hydroxyapatite (CHA) ceramics for clinical application [5–8].

We have previously reported a coralline hydroxyapatite/calcium carbonate (CHACC) material which shows promising clinical performance when implanted in sizes ranging from 10–100 × 10 × 10 mm^3^ [9, 10]. This material not only has properties such as porosity, surface structure, and osteoconductivity of coralline hydroxyapatite (CHA) as previously investigated [5–7] but also improves host tissue integration and can be completely biodegraded during bone remodelling [9]. Herein, we are focusing on smaller-sized CHACC, 200–300 *μ*m particles, with the potential to be injected facilitating administration for maxillofacial and dentistry applications.

To increase the functionality of bone biomaterials by hopefully contributing towards remodelling and host integration, stem cells are commonly added [11]. *In vitro* cellular 3D structures [12] created from stem cells resembling living tissue are known as organoids and have been developed as models for translational medicine [13] and gene therapy [14].

We have previously shown that human bone marrow (BM) mesenchymal stem cells (MSCs) can be cultured on CHACC [9, 10]. Human umbilical cord matrix (UCM) is a relatively new source of MSCs which has several advantages over BM including an abundant supply obtained noninvasively; it does not induce donor site morbidity and avoids ethical restrictions. Moreover, UCM MSCs have a higher proliferation rate, can be expanded further without loss of differentiation potential, and exhibit reduced immunogenicity for clinical use [15]. Osteogenesis of UCM-MSCs has been observed in both monolayer culture systems [16] and 3D culture systems, for example, on a demineralised bone [17] and polycaprolactone tricalcium phosphate [18]. Few studies have compared UCM MSC to BM MSC in 3D culture systems [16, 19].

Therefore, the aim of this study was to (1) confirm that MSCs could adhere to, proliferate on, and undergo osteogenic differentiation on 200–300 *μ*m CHACC particles to form 3D organoids and (2) to evaluate the osteogenic potential of UCM MSCs compared to BM MSCs. MSCs were isolated from BM and UCM and characterised by flow cytometry. CHACC was crushed into 200–300 *μ*m particles onto which MSCs were seeded and cultured in osteogenic differentiation medium. The resulting organoids were characterised by brightfield and scanning electron microscopy, alkaline phosphatase staining, and immunocytochemistry and PCR for key osteogenic markers.

## 2. Materials and Methods

### 2.1. Preparation of Human Bone Marrow

This study was approved by the South West Wales Research Ethics Committee (12/WA/0029) and all patients gave informed written consent. Exclusion criteria included preexisting conditions (e.g., connective tissue disease, diabetes, and malignancy) or medication (e.g., steroids and cytotoxic agents). BM aspirates were harvested from the iliac crest of two females (aged 25 and 29 years) and two males (aged 22 and 36 years) undergoing surgery to the pelvic ring or acetabulum using a BM aspiration needle (Mana-Tech Ltd., Burton-on-Trent, UK). Samples were collected in heparinised aspiration needles, transported at room temperature, and processed within 120 min.

Mononuclear cells (MNC) were isolated from the BM aspirate using Histopaque-1077 according to the manufacturer's instructions (Sigma-Aldrich, Poole, UK). Up to 40 × 10^6^ MNCs were seeded into 75 cm^2^ tissue culture flasks (CellSTAR, Greiner Bio-One, Stonehouse, UK) in 10 ml Minimum Essential Eagle Alpha Modification media with 10% fetal calf serum (Biosera, Uckfield, UK) and 1% Antibiotic-Antimycotic (100x, Life Technologies, Paisley, UK) and incubated for 7 days at 37°C under 5% CO_2_ in air. The media were changed every 3-4 days to remove contaminating nonadherent haematopoietic cells, and the adherent MSCs were cultured until 70% confluent. Cells were detached using Accutase according to the manufacturer's instructions (Sigma-Aldrich) and either propagated at a seeding density of 3 × 10^5^ per 75 cm^2^ culture flask or cryopreserved in 10% dimethyl sulfoxide (DMSO) (Sigma-Aldrich) in 90% FBS in liquid nitrogen for future use.

### 2.2. Preparation of Human Umbilical Cord Matrix

Human umbilical cords and placentas were collected from full-term births after elective caesarean section delivery of four mothers aged 30–35 and processed within 120 min. This study was approved by the South West Wales Research Ethics Committee and all mothers gave informed written consent. Inclusion criteria included mothers aged 18–50 who were at least 37 weeks pregnant. Exclusion criteria included preexisting health conditions (e.g., HIV, hepatitis C, or immunology complications), stillborn babies, or twins. A 3 cm section of the umbilical cord proximal to the placenta was dissected and the vasculature was carefully removed. The remaining matrix was finely diced using a scalpel. The diced tissue was placed in 25 cm^2^ flasks with 0.5 ml FBS. After 24 hours, 1 ml Dulbecco's modified Eagle's medium (DMEM, Life Technologies, Paisley, UK) supplemented with 10% FBS and 1% Antibiotic-Antimycotic was added. After 72 hours, an additional 3 ml DMEM was added. Cultures were subsequently fed twice weekly until 70% confluent at which point they were harvested and either propagated or cryopreserved *as above.* Cryopreserved UCM MSCs and BM MSCs were simultaneously used in the experiments for the assessment on CHACC.

### 2.3. Characterisation by Flow Cytometry

The following antibodies were used to phenotype the cells based on the International Society for Cellular Therapy (ISCT) criteria [20]: CD14-APC-eFluor780 (clone 61D3), CD34-eFluor450 (clone 4H11), CD73-FITC (clone AD2), CD90-APC (clone eBio5E10), CD105-PE (clone SN6) (eBioscience, Hatfield, Ireland, UK), CD19-PE-Cy7 (clone J3-119), and CD45-Krome Orange (clone J.33) (Beckman Coulter, High Wycombe, UK). All antibodies were mouse isotype IgG1, *κ*. Unstained cells were used as controls. Gating was performed on the forward and side scatter (FSC versus SSC) profile to remove debris and doublets based on scatter. Cells (3 × 10^5^) in 100 *μ*l FACS buffer (Dulbecco's PBS, Life Technologies; 0.2% BSA and 0.05% sodium azide, Sigma-Aldrich) were incubated on ice in the dark for 30 min with predetermined titrations of antibody. The cells were washed in FACS buffer and resuspended in 200 *μ*l FACS buffer for analysis.

The stained cells were analysed within 2 hours using a BD FACS Aria I flow cytometer with FACS Diva 6.1.3 software (BD Bioscience, Oxford, UK). The instrument was turned on for at least 1 hour prior to each run to allow the lasers to warm up, and Cytometer Setup & Tracking Beads (BD Bioscience) were used to check instrument performance. 10,000 cell events were recorded for each antibody. Voltages were set on unstained samples [21]. The FCS files were analysed in Kaluza 1.2 (Beckman Coulter) and the median fluorescent intensity (MFI) was displayed on logicle (biexponential) axes [22]. To convey information as to the density of events, contour density plots with visualised outliers were chosen as the standard plot [23].

### 2.4. Preparation of Coralline Hydroxyapatite/Calcium Carbonate Microscaffolds

CHACC (Affiliated Hospital, Hainan Medical College, Haikou, People's Republic of China) was crushed with a mortar and pestle and subsequently sieved through a 300 *μ*m followed by a 200 *μ*m sieve to capture only 200–300 *μ*m particles. The sieved CHACC were then autoclaved for sterilisation. 10 *μ*l of complete organoid medium (*α*-MEM supplemented with 10% FBS and 1% Antibiotic-Antimycotics), and three CHACC particles were placed into each well of a Terasaki microplate (Greiner Bio-One, Stonehouse, UK) and incubated for 24 hours at 37°C under 5% CO_2_ in air.

### 2.5. Formation and Differentiation of Organoids

3000 MSCs (P3-5) per 10 *μ*l organoid medium were added to the previously prepared scaffold particles in the Terasaki microplates to produce organoids. The next day, the organoids were transferred to a 100 mm petri dish (Fisher Scientific, Loughborough, UK) and swirled to allow the particles to come into contact with each other, enabling a larger scaffold conglomerate to be created. The organoids were treated with either plain organoid medium (control) or organoid medium supplemented with 100 nM dexamethasone, 10 mM *β*-glycerol phosphate, and 100 *μ*M 2-phosphate-ascorbic acid to stimulate osteogenic differentiation.

### 2.6. Characterisation by Microscopy and Live/Dead Assay

The organoids were analysed by brightfield and scanning electron microscopy (SEM). The samples were analysed on days 1, 7, 14, and 21 during differentiation. For SEM, the samples were cut to 1 mm diameter, fixed in 4% glutaraldehyde (Sigma-Aldrich), and then gradually dehydrated through an ethanol series, using sequentially higher concentrations of ethanol (70%, 80%, 90%, 95%, and 100%) for 10 min each. Finally, samples were further dehydrated in 50% hexamethyldisilazane (Sigma-Aldrich) diluted with ethanol for 10 min, followed by full immersion in absolute hexamethyldisilazane, and left to evaporate overnight in a fume cupboard. Control scaffold particles (no cells) were also incubated for 7 days in organoid medium, at 37°C under 5% CO_2_ in air, before being fixed and dehydrated. SEM was carried out using a Hitachi S-4800 II SEM with an accelerating voltage of 1 kV at 110x and 5000x. Samples were mounted onto SEM stubs using a double-coated carbon conductive tape (Acros Organics, supplied by Fisher Scientific).

Cell viability was also assessed after 14 and 21 days using Live/Dead assay kit (Life Technologies, Paisley, UK), in which cells were stained with calcein acetoxymethyl (0.1 *μ*g/ml) and propidium iodide (1 *μ*g/ml) and viewed by fluorescence microscopy.

### 2.7. Assessing Differentiation by Immunocytochemistry

At 21 days differentiation, organoids were washed in PBS and orientated within a large droplet of Bright Cryo-M-Bed embedding compound which was snap frozen on dry ice. 10 *μ*m sections were then cut using a Leica CM1900 and melted onto slides for staining. Slides were fixed in 10% neutral buffered formalin (Sigma-Aldrich), permeabilised in PBS : 0.1% Triton X-100 (Sigma-Aldrich), and incubated in PBS : 0.5% bovine serum albumin (BSA) (Sigma-Aldrich) for 30 min to block nonspecific binding. The organoids were stained with preoptimised concentrations of monoclonal mouse primary antibodies targeting Runx2 (3 *μ*g/ml) (R&D Systems, Abingdon, UK), osteocalcin (10 *μ*g/ml), or polyclonal rabbit primary antibody targeting type I collagen (10 *μ*g/ml) (Abcam, Cambridge, UK) overnight in a humidified environment at +4°C. Unbound primary antibody was removed by washing in PBS and the slides were incubated for 1 hour in the dark at room temperature with a secondary NL557-conjugated anti-mouse or NL493-conjugated anti-rabbit antibody (1 : 200) (R&D Systems). Slides were washed in PBS and stained with 0.1% 4′,6-diamidino-2-phenylindole (DAPI, Life Technologies) for 1 min at room temperature. Each sample was washed in PBS and imaged using a confocal microscope (Zeiss LSM710, Oberkochen, Germany). Negative (no primary antibody) and blank (scaffold without cells) controls were included.

### 2.8. Alkaline Phosphatase Staining

The slides were fixed in ice cold 70% ethanol (Fisher Scientific) for 10 min after sectioning and washed in PBS, before being immersed in ALP substrate kit (Vector Laboratories, Peterborough, UK) according to the manufacturer's instructions. Slides were stained in 0.1% DAPI (Life Technologies) for 1 min at room temperature to stain nuclei and imaged using a confocal microscope.

### 2.9. Real-Time PCR Analysis

Key markers of osteogenesis (Runx2, ALP, and type I collagen) were assessed using real-time PCR. 21 days post differentiation, RNA was isolated from the cells using the MasterPure kit (Cambio, Cambridge, UK) according to the manufacturer's instructions. In brief, samples were lysed in 300 *μ*l of tissue and cell lysis solution at 65°C for 30 min. Protein was precipitated out of solution using 150 *μ*l of protein precipitation reagent. After centrifugation, the supernatant was collected and incubated for 1 hour at 37 °C with deoxyribonuclease to remove DNA. This process was then repeated. RNA was collected by precipitation using 2-propanol and centrifugation. 1 *μ*g of RNA was reverse transcribed into cDNA using RETROscript® Kit (Life Technologies) in a reaction volume of 20 *μ*l. Real-time PCR reactions were run at 50°C for 2 min and 95°C for 2 min, as an initial denaturation step, followed by 40 cycles at 95°C for 15 secs to denature and 60°C for 30 secs to anneal using SsoFast EvaGreen Supermix and the 2005 MyiQ real-time PCR detection system (Bio-Rad Laboratories, Hemel Hempstead, UK). Amplification of each gene included 10 *μ*l of SsoFast EvaGreen®, 0.6 *μ*l forward primers, 0.6 *μ*l reverse primers, 6.8 *μ*l of nuclease free water, and 2 *μ*l of diluted cDNA (1 : 10 with nuclease-free water). This was followed by a melt curve analysis. The cycle threshold (CT) of amplification for each gene of interest (Table 1) was normalised against the housekeeping gene GAPDH in all samples, and relative gene expression level was determined by the 2^(GAPDH CT-Test CT) method.

### 2.10. Statistical Analysis

All experiments were repeated at least once until consistent results were obtained. Nonparametric tests were used to analyse statistical data (Statistica Version 6, StatSoft Ltd., UK). All data are expressed as mean ± standard error.

## 3. Results

### 3.1. Characterisation by Flow Cytometry

**Figure 1:**
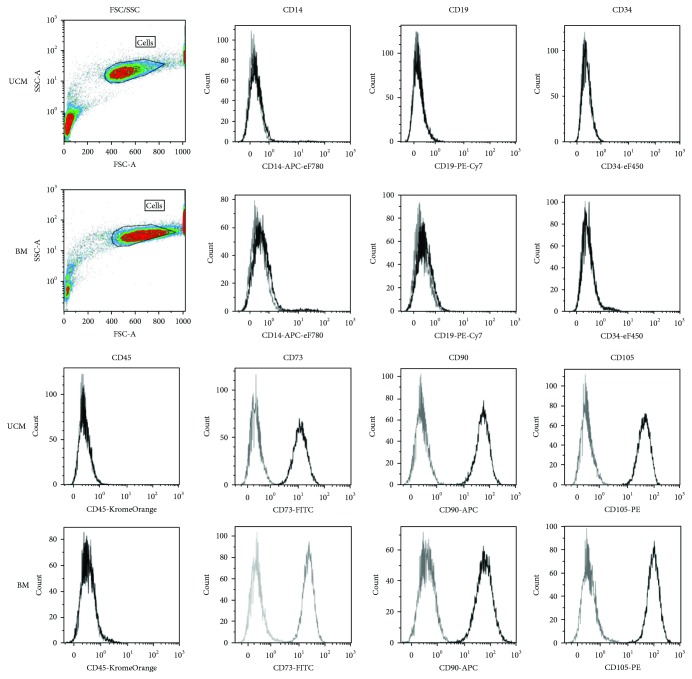
Human UCM and BM MSCs single stained with antibodies against surface markers. MSCs were CD14-CD19-CD34-CD45-CD73+CD90+ and CD105+, correlating with an MSC phenotype defined by ISCT. Grey: unstained; black: stained.

UCM and BM MSC demonstrated the traditional MSC phenotype [20] showing positive expression for CD73, CD90, and CD105 and negative expression for the haematopoietic markers CD14, CD19, CD34, and CD45 (Figure 1).

### 3.2. Morphology Characterised by Bright Field and Scanning Electron Microscopy

**Figure 2:**
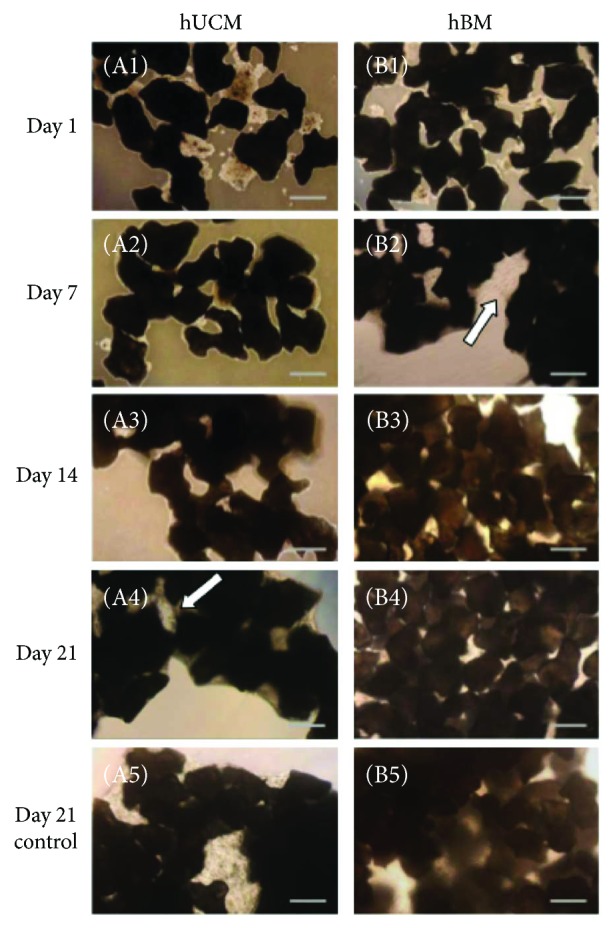
Bright field images of organoids with UCM (A) and BM (B) MSCs during the process of osteogenesis. Images were taken on days 1 (A1, B1), 7 (A2, B2), 14 (A3, B3), and 21 (A4, B4) of the differentiation process and compared to a control (*α*-MEM) image at day 21 (A5, B5). BM MSCs were observed to proliferate into the voids created by the numerous coral particles by day 7 and UCM MSCs by day 7 (white arrows). SP: scaffold particle. Scale bars: 250 *μ*m.

**Figure 3:**
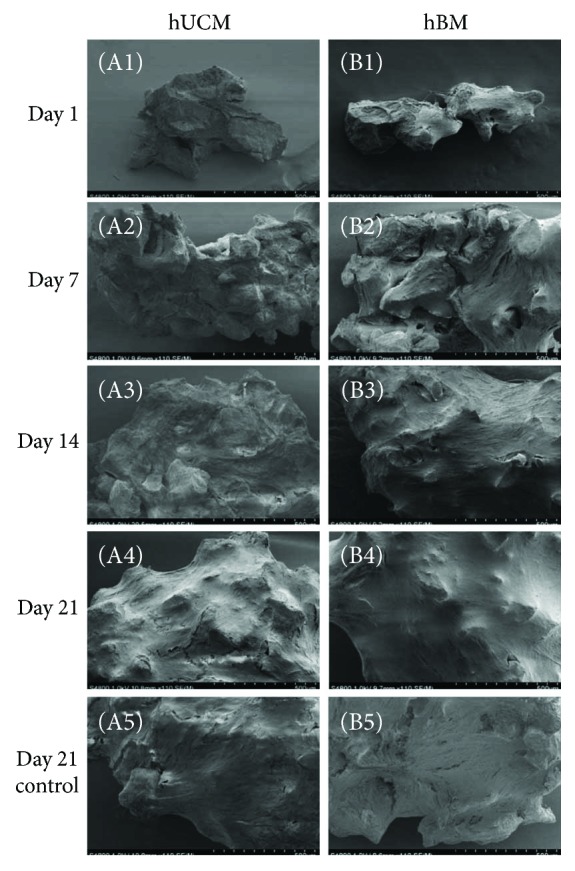
SEM images of organoids with UCM (A) and BM (B) MSCs during the process of osteogenesis. Images were taken on days 1 (A1, B1), 7 (A2, B2), 14 (A3, B3), and 21 (A4, B4) of the differentiation process and compared to a control (*α*-MEM) image at day 21 (A5, B5). Over time, the voids within the organoid were filled with MSCs and associated extracellular matrix which formed a smoother surface that covered CHACC surfaces. Significant coverage was exhibited using BM MSCs at day 14 (B3), whereas UCM MSCs did not cover surfaces until day 21 (A4). Scale bars: 500 *μ*m.

Bright field (Figure 2) and SEM images (Figure 3) were taken on days 1, 7, 14, and 21 of the differentiation process and compared to a control image taken at day 21 with no addition of osteogenic supplement. Bright field images (Figure 2) showed large gaps in between particles devoid of UCM MSCs (Figure 2(A4), white arrow) until day 21. In contrast, BM MSCs (Figure 2(B)) were observed to proliferate and rapidly occupy the spaces between particles as early as day 7 and did not change in appearance after this time point. SEM images (Figure 3) showed over time the surface of the organoids to become smoother due increasing cell density and deposition of extracellular matrix, which bound the CHACC particles together forming a larger conglomerate. Smoother surfaces were exhibited using BM MSCs (Figure 3(B3)) at day 14, whereas UCM MSCs (Figure 3(A4)) did not cover the entire surface until day 21. The addition of osteogenic supplement was not observed to have any significant impact on organoid morphology in relation to the control images at 21 days.

Cell viability was also assessed after 14 and 21 days using Calcein acetoxymethyl (0.1 *μ*g/ml) and propidium iodide (1 *μ*g/ml) and viewed by fluorescence microscopy. Dead cells were barely observed on CHACC using UCM or BM MSCs, which demonstrated that the cell viability was not affected by CHACC microparticles.

### 3.3. Osteogenesis Characterised by Immunocytochemistry and Alkaline Phosphatase Staining

**Figure 4:**
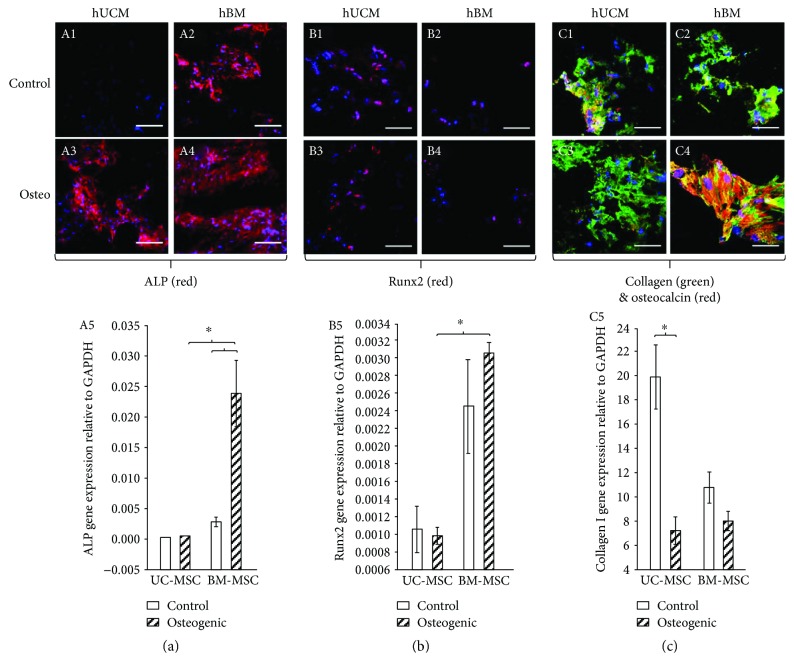
On day 21, control and osteogenically induced organoids were cryosectioned (10 *μ*m) and stained for alkaline phosphatase (ALP) (a), Runx2 (b), collagen type I, and osteocalcin (c). Real-time PCR was used to assess mRNA levels of ALP (A5), Runx2 (B5), and collagen type I (C5) in UCM and BM MSCs lysed directly from the control and osteogenically induced scaffolds at 21 days. MSCs derived from UCM expressed good collagen I mRNA and protein production but poor ALP and Runx2 in relation to MSCs derived from BM. *n* = 4 (^∗^*p* < 0.05).

UCM and BM MSCs were observed throughout the organoid by 21 days of differentiation. Excluding UCM MSCs grown in control *α*-MEM that did not label for ALP (Figure 4(a), A1), osteogenically induced and noninduced organoids were positive for ALP (Figure 4(a), A2–A4), Runx2 (Figure 4(b), B1–B4), type I collagen, and osteocalcin (Figure 4(c), C1–C4). ALP was active within the MSC's cytoplasm. Runx2 was largely found within the nucleus of MSCs and appeared purple due to blending with the nuclear stain DAPI. Type I collagen and osteocalcin were found within the extracellular matrix on the surface of the organoid.

ALP (Figure 4(a), A5), Runx2 (Figure 4(b), B5), and type I collagen (Figure 4(c), C5) were also assessed by real-time PCR. Osteogenically induced BM MSCs were shown to have significantly more ALP (Figure 4(a), A5) and Runx2 (Figure 4(b), B5) mRNA than osteogenically induced UCM MSCs, relative to the housekeeping gene, GAPDH. However, control BM MSCs were also shown to have significantly more Runx2 mRNA compared to UCM MSC. Nonosteogenically induced UCM MSCs showed high type I collagen mRNA and protein expression but low ALP and Runx2.

## 4. Discussion

Novel biomaterials for bone regeneration are desired as they do not have inherent disadvantages of autografts (blood loss, infection and morbidity risks, and limited supply). However, they need to match the gold standard of autografts in bone replacement surgery. CHACC has been shown to have excellent properties to function as a bone graft, but it lacks the key component required for autografts: living cells with osteogenic capacity.

In this study, UCM and BM MSCs were incorporated with CHACC microparticles to form organoids and their *in vitro* osteogenic potential was assessed and compared. Human UCM MSCs have been proven to differentiate down the osteogenic lineage and share common surface markers to BM MSCs [15]. CHACC is already used as a bone graft. Therefore, it was expected to provide a 3D structure for UCM and BM MSC attachment, proliferation, and differentiation. Organoids can result in increased cell numbers compared to the cell suspension method [24], and the hydroxyapatite layer on CHACC should accelerate the differentiation of cells and consequently mineralisation [24–26].

Although both BM and UCM MSCs were able to attach to CHACC microparticles, form organoids, proliferate, and differentiate down the osteogenic lineage, as expected, the BM MSCs showed higher levels of osteogenic differentiation than UCM MSCs. BM MSCs showed a dramatic increase in cell proliferation indicating that they entered into the first stage of osteogenic differentiation [27] before UCM MSCs. Increased osteogenic differentiation in BM MSCs was further evidenced by increased expression of runx2 and ALP and the labelling of osteocalcin in immunocytochemistry [25]. Other researchers have also found BM MSCs to have superior osteogenic potential compared to UCM MSCs [28]. Similar to our study, Schneider et al. found BM MSCs to express more ALP but less type I collagen than UCM MSCs [29]. Zhang et al. compared MSCs on 3D scaffolds derived from different sources and found BM MSCs to form more bone than UCM MSC [18]. Reduced osteogenic potential of UCM MSCs maybe explained by the anatomical origins that they were derived from. Panepucci et al. found higher levels of genes related to osteogenesis in BM MSCs compared to umbilical cord vein MSCs [30]. Umbilical cord vein MSCs expressed genes more related to matrix remodelling via metalloproteinases and angiogenesis. Consequently, UCM MSCs could be less committed to osteogenesis but instead committed to angiogenesis.

Interestingly, Zhang et al. also showed that UCM MSCs could differentiate down the osteogenic lineage better than BM MSCs if they were cultured in monolayer [18]. Based on this study, future work should investigate differentiating UCM MSCs in monolayer first and then seed these cells onto 3D scaffolds. Although ectopic bone formation using UCM MSCs has been proven to be inferior compared to BM MSCs in vivo, the angiogenic nature of UCMs has been utilised to improve bone regeneration. Todeschi et al. showed that UCM MSCs implanted orthotopically caused a similar amount of new bones to form compared to BM MSCs by recruiting host osteogenic cells [31]. Chen et al. also utilised the angiogenic nature of umbilical cord mesenchymal stem cells by coculturing them with human umbilical cord vein endothelial cells. They found that a similar amount of new bone formation could be achieved in vivo compared to coculturing human umbilical cord vein endothelial cells with BM MSCs [32].

There are a number of limitations to this study; immunocytochemistry and real-time PCR only assess a narrow spectrum of markers, meaning MSCs could be differentiating down a lineage not being specifically looked at. Furthermore, quantitative PCR only gives a snap shot of RNA expression at the day it is extracted and has a very short half-life of approximately 9 hours [33] meaning expression levels could be missed. Cell attachment and growth was limited to arbitrary qualitative assessment via bright field and SEM images.

Future work is expected to overcome the inferior osteogenic capacity of UCM MSCs, such as to initiate the osteogenic differentiation at 2D culture stage, and the osteogenic gene and protein expression will be assessed at more time points to show the full spectrum of differentiation over the three-week period or longer to assess the full potential of UCM MSCs. Also, to utilise the angiogenic nature of UCM, MSCs with CHACC should be explored as well.

## 5. Conclusion

This study has shown that 200–300 *μ*m CHACC granules can be a suitable carrier for human MSC proliferation and differentiation *in vitro*, for the purpose of injectable delivery opening up the use of CHACC for new applications within maxillofacial surgery and dentistry. In addition, UCM MSCs show inferior osteogenic capacity when cultured as CHACC organoids compared to BM MSCs. Therefore, further research is required to optimise the osteogenicity of UCM MSCs to release their full potential as a readily available, accessible, and abundant tissue source for bone tissue engineering.

## Figures and Tables

**Table 1:** Primer sequences used in real-time PCR.

Gene	Forward primer (5′-3′)	Reverse primer (5′-3′)
Glyceraldehyde-3-phosphate dehydrogenase	TCA TTG ACC TCA ACT ACA TGG T	TCT CGC TCC TGG AAG ATG GTG
RUNX2	CCT AGG CGC ATT TCA GGT GCT T	CTG AGG TGA CTG GCG GGG TGT
Type I collagen	ATG TTC AGC TTT GTG GAC CTC CGG	CGC AGG TGA TTG GTG GGA TGT CT
Alkaline phosphatase	GAC CCT TGA CCC CCA CAA T	GCT CGT ACT GCA TGT CCC CT

## Data Availability

Data are available from Swansea University; requests for access should be made to Dr. Zhidao Xia (z.xia@swansea.ac.uk).
